# Assessing the Risk of Exotic Mosquito Incursion through an International Seaport, Newcastle, NSW, Australia

**DOI:** 10.3390/tropicalmed6010025

**Published:** 2021-02-17

**Authors:** Cameron E. Webb, Philippe G. Porigneaux, David N. Durrheim

**Affiliations:** 1 Marie Bashir Institute of Infectious Diseases and Biosecurity, University of Sydney, Westmead, NSW 2006, Australia; 2 Medical Entomology, NSW Health Pathology, Westmead Hospital, Westmead, NSW 2145, Australia; 3 Hunter New England Population Health, Wallsend, NSW 2287, Australia; Philippe.Porigneaux@health.nsw.gov.au (P.G.P.); David.Durrheim@health.nsw.gov.au (D.N.D.); 4 School of Medicine and Public Health, University of Newcastle, Callaghan, NSW 2308, Australia

**Keywords:** *Aedes notoscriptus*, *Aedes aegypti*, *Aedes albopictus*, water-holding containers, exotic mosquito surveillance, urban ecology

## Abstract

Exotic mosquitoes, especially container-inhabiting species such as *Aedes aegypti* and *Aedes albopictus*, pose a risk to Australia as they bring with them potentially significant pest and public health concerns. Notwithstanding the threat to public health and wellbeing, significant economic costs associated with the burden of mosquito control would fall to local authorities. Detection of these mosquitoes at airports and seaports has highlighted pathways of introduction but surveillance programs outside these first ports of entry are not routinely conducted in the majority of Australian cities. To assist local authorities to better prepare response plans for exotic mosquito incursions, an investigation was undertaken to determine the extent of habitats suitable for container-inhabiting mosquitoes in over 300 residential properties adjacent to the Port of Newcastle, Newcastle, NSW. More than 1500 water-holding containers were recorded, most commonly pot plant saucers, roof gutters, and water-holding plants (e.g., bromeliads). There were significantly more containers identified for properties classified as untidy but there was no evidence visible that property characteristics could be used to prioritise property surveys in a strategic eradication response. The results demonstrate that there is potential for local establishment of exotic mosquitoes and that considerable effort would be required to adequately survey these environments for the purpose of surveillance and eradication programs.

## 1. Introduction

Mosquito-borne disease is a concern for health authorities across Australia. While pathogens, such as Ross River (RRV) and Barmah Forest viruses (BFV), transmitted by endemic mosquitoes result in approximately 5000 notifications of illness each year [1,2], there is increasing concern regarding the introduction of exotic mosquitoes that may transmit pathogens of serious health concern such as dengue (DENV), chikungunya (CHIKV), and Zika (ZIKV) viruses [3]. Existing mosquito surveillance programs of local health authorities are focused on endemic vectors and pathogens while exotic mosquito surveillance is limited to areas within airports and seaports, where international aircraft and vessels arrive. The exotic mosquitoes *Aedes aegypti* L. (Diptera: Culicidae) and *Aedes albopictus* Skuse (Diptera: Culicidae) both pose substantial pest and public health concerns [4,5,6,7]. There is a need to develop response plans for exotic mosquitoes [3] and this requires an understanding of local risk factors within local regions.

There are multiple pathways of potential introduction of exotic mosquitoes into Australia. Increasing international travel is a substantial risk factor, not only the number of flights arriving in Australia each year but also the steady growth in airports around the country receiving international flights. There has been an increase in Australian travellers returning from overseas infected with exotic mosquito-borne diseases [8]. This is especially the case for DENV infections, with a steady growth in cases reported from 2004 to 2013, the majority of which originate from Indonesia [9]. Molecular analysis of exotic mosquitoes intercepted at Australian airports and seaports identified the most likely pathways of introduction being flights from Indonesia [10]. While international airports, and associated freight-handling facilities, are the highest risk entry points for exotic mosquitos, the importance of seaports should not be underestimated and exotic mosquitoes have been reported from international seaports in both Australia and New Zealand [10,11,12,13]. A review of 244 suspected exotic mosquito interceptions at seven airports, six seaports, and their transitional facilities in New Zealand identified *Ae. aegypti* and *Ae. albopictus* as risks and 66% of known interceptions were at six New Zealand seaports [14]. 

The container-inhabiting mosquito *Ae. albopictus* is also of concern, especially given its detection in Torres Strait Islands [15], and its reputation as one of the most invasive pest mosquitoes internationally [16,17]. Modelling has suggested a temperate climate-tolerant strain of this mosquito could become established in coastal regions of eastern Australia as far south as Victoria [5]. Recent studies have indicated that while the pathways of entry of these two mosquitoes differ [18], the regular detection of these mosquitoes at first ports of entry into Australia highlight the need for local authorities to develop response plans to local incursions of these mosquitoes. 

Urban environments in Australia provide many opportunities for *Ae. aegypti* and *Ae. albopictus* with preferred oviposition and larval development sites being natural and artificial water-holding containers. These habitats can be diverse and range from tree-holes and water-holding plants (e.g., bromeliads) to bird baths, rainwater tanks, roof guttering and pot plant saucers. The larval habitats of these mosquitoes have been well studied in Australia, where *Ae. aegypti* has posed a significant threat transmitting DENV in central and far north QLD [19]. There have also been many studies in Australia documenting the habitat associations of the endemic container-inhabiting mosquito *Ae. notoscriptus*. This mosquito is a widespread nuisance-biting pest and while it is not considered a DENV vector, it can effectively transmit RRV and BFV. Research focused in metropolitan areas of Brisbane and Perth has highlighted the importance of *Ae. notoscriptus* as a nuisance-biting pest, but not as significant a pest species as *Ae. albopictus*, and identified a diverse categories of water-holding containers utilized by this mosquito and, potentially, by *Ae. aegypti* or *Ae. albopictus* [6,20,21,22]. 

The Australian Government Department of Agriculture, Water and the Environment (DAWE) has an exotic mosquito surveillance program within 400 m of international berths at airports and seaports around Australia in accordance with World Health Organization (WHO) International Health Regulations [23]. In recent years, there has been a steady increase in the number of exotic mosquitoes, including *Ae. aegypti* and *Ae. albopictus*, detected in first ports of entry across Australia [10,15,18]. Coupled with the southern movement of *Ae. aegypti* in QLD, potentially aided through road-based freight transport [13], and given the ever increasing human movement and freight transport across the country, there remains the potential for introduction of *Ae. aegypti* from QLD into the local region. There are no established populations of *Ae. albopictus* on mainland Australia but these have been reported from, and actively managed in, the islands of the Torres Strait [5].

Exotic mosquitoes have been detected at Australian air and seaports in recent years [10,15] including freight-handling facilities (i.e., approved premises) that sit outside the normal footprint of international ports. Concerns have been raised regarding these facilities as they do not provide a harsh environmental buffer more typically found surrounding airports that limits the likely dispersal of mosquitoes away from vessels or freight. Of particular concern are residential and industrial developments immediately adjacent to seaports that provide a potentially suitable habitat for mosquitoes, both adult refuge and suitable larval habitats.

The Newcastle and broader Hunter New England region of NSW experiences annual transmission of endemic mosquito-borne pathogens as well as imported cases of exotic mosquito-borne disease. Over the twenty years to December 2019, 4092 cases of RRV, 1495 cases of BFV, and 317 cases of DENV were reported in residents of Hunter New England Local Health District [24]. A smaller number of imported cases of CHIKV [12] and malaria 221 have also been reported in recent years, while no cases of ZIKV have been reported [24,25]. While some debate surrounds the potential role of a changing climate in future mosquito-borne disease risk [26], it is unlikely that there will be a substantial increase in the public health risks associated with local mosquito populations based on changes in temperature, rainfall, and sea level rise alone. However, the introduction of invasive exotic container-inhabiting mosquitoes could significantly change local pest and public health risks and there are actual and potential pathways of introduction of exotic mosquitoes into the region, as well as pathogens as evident in the identified cases of mosquito-borne pathogen infection in returning travellers. 

The most important mosquito species of pest and public health concern in the region are *Aedes vigilax* Skuse (Diptera: Culicidae), a mosquito closely associated with estuarine wetlands; *Culex annulirostris* (Skuse) (Diptera: Culicidae), a mosquito associated with freshwater wetlands; and *Aedes notoscriptus* (Skuse) (Diptera: Culicidae), a mosquito closely associated with water-holding containers in urban areas [27]. While, historically, mosquito species capable of transmitting DENV, such as *Ae. aegypti*, were present in the local region, the mosquito has had a restricted range in Australia since the 1950s and is currently only found in central and far north QLD [4,28]. There is some speculation that increasing temperatures associated with predicted climate change may directly or indirectly facilitate a return of *Ae. aegypti* in more temperate regions of Australia [29,30] and so may be a future concern with the possible transport of this mosquito, or its eggs deposited on personal belongings (e.g., garden ornaments, pet water bowls, vases), being moved from QLD into the local area, as has occurred in Europe [31]. Road transport has also been identified as a pathway of movement of *Ae. aegypti* from coastal far north QLD to the inland township of Tennant Creek in the Northern Territory [13].

The Port of Newcastle is adjacent to the residential suburb of Carrington, NSW. It represents a unique setting for managing exotic mosquito threats given that many residential and industrial properties are situated outside the Port remain within the 400 m zone of international berths and well within the estimated flight range of *Ae. aegypti* or *Ae. albopictus* that is generally considered to be approximately 200 m but in some circumstance may exceed this distance [32,33]. While no exotic mosquitoes have been detected by the local surveillance program, given the relatively more suitable conditions provided by the suburban landscape, as opposed to the Port of Newcastle itself, concern has been raised by local authorities that this area could be at high risk of exotic mosquito establishment. In an assessment of Australian air and seaports [34], the Port of Newcastle was identified as having a moderate risk of exotic mosquito incursion due to the potential of vessels carrying water-holding containers containing the eggs and/or larvae, as well as adults, of exotic mosquitoes. With the need to develop responses to actual and potential exotic mosquito incursions in NSW [3], this area was considered a suitable location to investigate the potential for *Ae. aegypti* or *Ae. albopictus* to become established and consider the appropriate responses of local authorities.

The aim of this investigation was to provide an assessment of exotic mosquito risk in suburban residential areas adjacent to the Port of Newcastle by reviewing relevant literature on current and projected risks of exotic container-inhabiting mosquitoes and undertaking a survey of actual and potential mosquito habitats, as assessed using the proxy of the endemic container-inhabiting mosquito, *Ae. notoscriptus*, within a representative sample of residential properties. 

## 2. Materials and Methods

### 2.1. The Study Site

**Figure 1:**
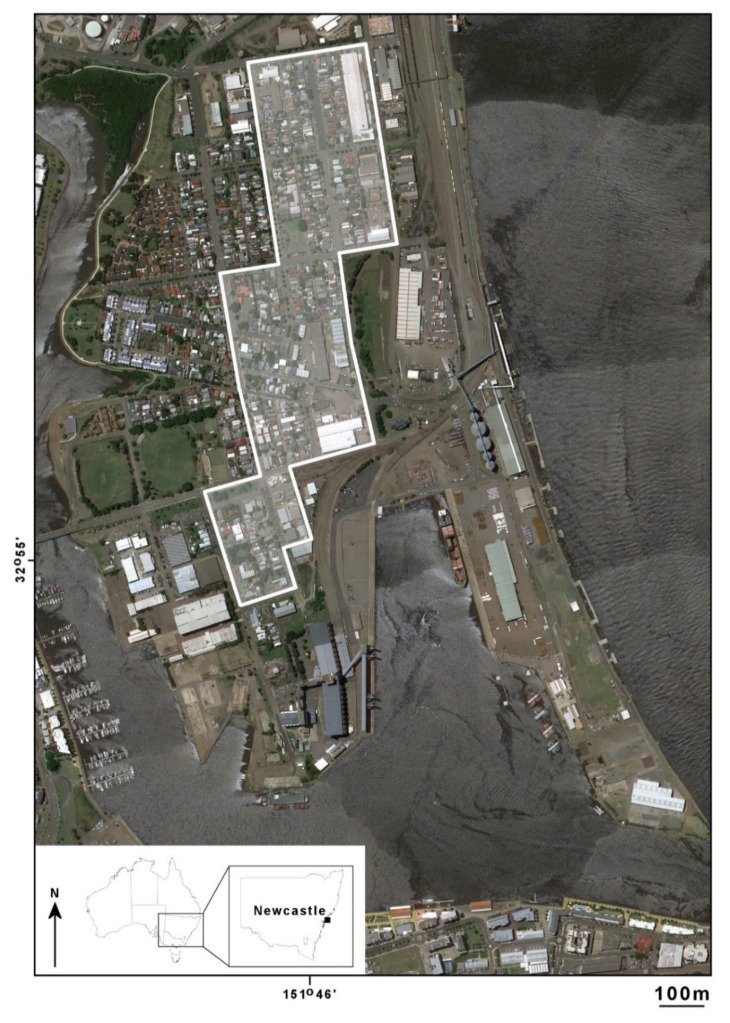
Residential and industrial areas where property surveys were conducted within the suburb of Carrington, adjacent to the Port of Newcastle, NSW, Australia. (Image: Google Earth; accessed 28 August 2020.).

Mosquitoes and mosquito habitats were surveyed in the suburb of Carrington (NSW, Australia), located in the city of Newcastle, the second largest metropolitan region of NSW, containing a mix of residential, commercial and industrial properties, with a residential population of approximately 2000 people [35]. The suburb was selected as it is adjacent to the Hunter River and in close proximity to the Port of Newcastle. In 2019, there were 2296 vessel movements through the Port—the majority of these were associated with coal exports with over 60% of movement between Newcastle and Japan or China [36]. There is a marked variation in the style of residential property ranging from stand-alone dwellings on relatively large blocks of land to small, high-density dwellings, terrace houses and unit blocks. Generally, the suburb is heavily urbanised with minimal open space or parklands. Based on an assessment of aerial photographs, a section of Carrington was selected for house-to-house surveys. The study site (Figure 1) was located within or adjacent to the 400 m zone of vessel berths at the Port of Newcastle and within known potential flight ranges (approximately 150 m) of *Ae. aegypti* or *Ae. albopictus*. 

### 2.2. Mosquito Population and Habitats Surveys

Surveys were conducted on four days, 3–8 March 2016. The majority of surveys were conducted by two researchers (Webb and Porigneaux) who had previous mosquito surveillance experience. The study site was systematically surveyed with details recorded for each property, irrespective of whether residents were present or not. Where residents were present, they were asked for permission to inspect their property for actual or potential mosquito habitats. Participating residents were provided with a fact sheet prepared by Hunter New England Population Health that outlined the project objectives together with information on local mosquitoes and personal protection measures. For properties where a resident was not present, the property was inspected through observations made from the street.

Each property was assessed according to a number of key characteristics known to be associated with increased risk of mosquito suitability. These characteristics were based on “generic property inspection template” criteria provided by Queensland Health [37] in the Queensland Dengue Management Plan, 2015–2020, with reference to the Premise Condition Index (PCI) developed by Tun-Lin [38] but modified for local conditions. Characteristics recorded included the percentage of shade over the property (<25%, 25–50%, >50%), the state of the property (tidy (i.e., lawn neat, no rubbish), intermediate and untidy (i.e., overgrown vegetation, accumulation of rubbish and other items)) and condition of the dwelling (well maintained, intermediate, dilapidated). 

In the initial survey stages, properties were inspected together by researchers and, for each property, agreement was reached on property categorization. This enabled consistency throughout the remaining surveys that were conducted individually by researchers. However, where uncertainty existed for individual properties, researchers consulted and agreed on categorization. Where property owners were not present, only visual surveys of front yards were undertaken. Researchers did not enter properties without the permission of residents. Where property owners were present, and permission granted to enter, properties were surveyed for the presence of actual and potential water-holding containers. The number of containers identified was recorded for 15 pre-determined categories based on previously published studies on container-inhabiting mosquitoes in Australia [39]. Where water-holding containers of a category not included on data sheets were detected, additional notes were included on the data sheet. The total number of containers in each category was recorded along with the presence of any mosquito larvae or pupae. Mosquito larvae or pupae were sampled either using a standard 300 mL dipper (Australian Entomological Supplies, Bangalow, NSW), or disposable pipettes. For smaller containers, the contents were emptied into a shallow plastic tray for ease of picking specimens. Mosquito larvae were collected, preserved in 70% ethanol, and returned to the laboratory for identification while pupae were collected and returned to the laboratory and placed in specialised containers (Australian Entomological Supplies, Bangalow, NSW) to allow emergence. Immature specimens were identified according to the taxonomic key of Russell [40] and pictorial guide of Webb et al. [27]. Climatic data for the survey period were obtained from the Bureau of Meteorology (Station ID: 061055, Newcastle Nobbys Signal Station). For each of the property classifications, the mean number of containers was calculated and one-factor analysis of variance (ANOVA) was used to determine the significance of any differences.

## 3. Results

Climatic conditions leading up to and during the survey were not ideal for mosquito surveys given the prevailing hot and dry conditions. Temperatures during February 2016 were slightly higher (mean daily minimum temperature 21.0 °C; mean daily maximum temperature 26.4 °C) than the long-term average (mean daily minimum temperature 19.4 °C; mean daily maximum temperature 25.4 °C) while total monthly rainfall for February 2016 was 37.0 mm compared to the long-term average for February of 107.6 mm. The substantially lower rainfall was likely to influence the prevalence of suitable conditions for mosquitoes during this survey in March 2016 with smaller containers more likely to be dry. Given the dry conditions, survey results were not likely to provide an accurate measure of the productivity of individual containers or allow for comparisons between average productivity per container based on property characteristics. For this reason, the abundance of immature mosquitoes was not recorded but samples of larvae and pupae collected were retained, sorted by property and identified to confirm species.

A total of 337 properties were inspected from the street front with only 22/337 properties having no access (i.e., high fences, dense vegetation) to undertake any visual assessment. There was substantial diversity in dwelling types across the suburb, making assessment of actual and potential mosquito habitats difficult. In some cases, the backyard of properties were only accessible through the dwelling itself (e.g., the front door to dwelling was located on the immediate street front), and this created barriers to both visual and actual inspection for water-holding containers. There were a number of large industrial properties in the area that contained potential mosquito habitats but these were not surveyed as the focus of this study was residential properties and access to industrial properties could have resulted in additional occupational safety and health concerns. It was also observed that there was substantial residential renovation and redevelopment underway across the suburb, with several properties being significantly renovated while others were being demolished for the purpose of property reconstruction. These properties provided potential mosquito habitats onsite (e.g., plastic sheeting covering building supplies, plastic buckets, discarded bottles and cans) but property surveys were not undertaken due to occupational safety and health concerns.

A total of 139 properties were recorded as being unattended by residents but 270/337 properties were able to be at least visually inspected for water-holding containers with 42/337 properties being inspected fully and 25/337 properties partially inspected. Access to property was denied by a resident present on three occasions, the reasons cited were related to privacy. It was noteworthy that in these small number of instances, the properties were assessed as potentially high risk due either to the overgrown nature of vegetation or accumulation of rubbish. Overall, the properties within the study area were generally considered relatively unsuitable for mosquitoes based on categorization of shading, tidiness and dwelling condition. A total of 284/337 properties had <20% shade, 263/337 were considered to be tidy and 305/337 were considered to be well maintained. 

Where property surveys could be undertaken, immature mosquitoes were only detected in 15 of the 69 properties inspected (i.e., where containers could be accessed to take water samples or visually inspect for mosquitoes). Despite this relatively low proportion of properties surveyed, there were a large number of individual containers identified (Table 1). Over 1500 containers were recorded, with the most common being pot plants (34.7%), roof gutters (13.6%), bromeliads (8.7%) and buckets (8.0%). Over 20.0% of containers detected were small, estimated to hold less than 2L of water, and ranged from discarded bottles, plastic containers and garden accoutrements. When considering the time taken to survey these properties, it was estimated that the average time was between 10 and 12 min. On some occasions, it took up to 18–20 min but the longer times required for surveys were often due to interaction with residents rather than any physical barriers restricting access to area of the property. 

Relatively few containers positive for immature mosquitoes were recorded. The low mosquito abundance was primarily considered due to the prevailing dry conditions and lack of water in the majority of individual containers surveyed. The immature stages of two mosquito species were recorded, *Ae. notoscriptus* and *Culex quinquefasciatus* Say (Diptera: Culicidae). No specimens of *Ae. aegypti* or *Ae. albopictus* were detected. 

**Figure 2:**
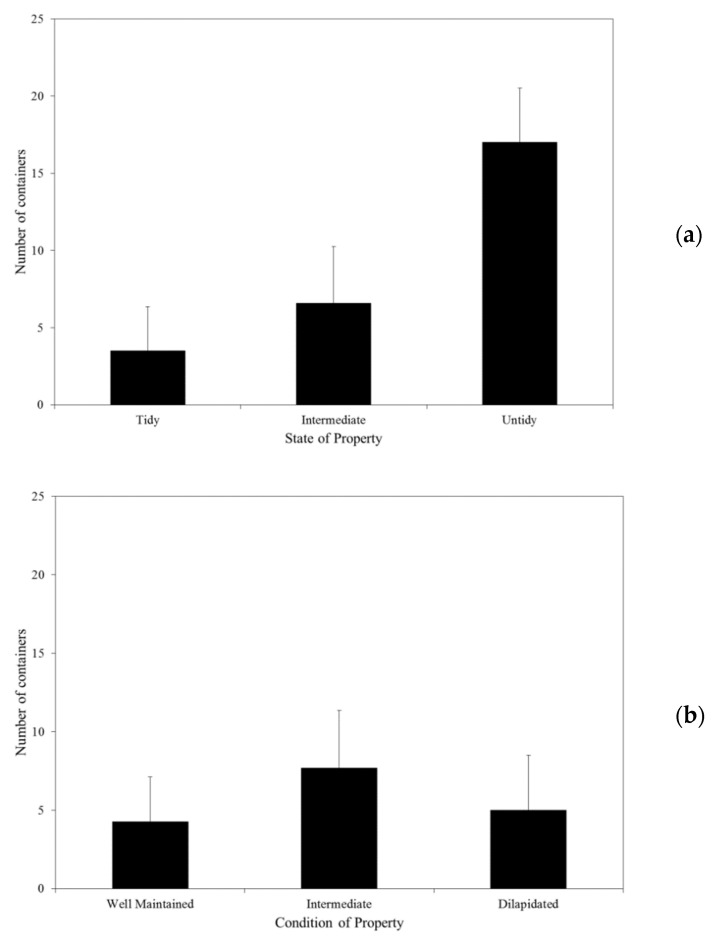

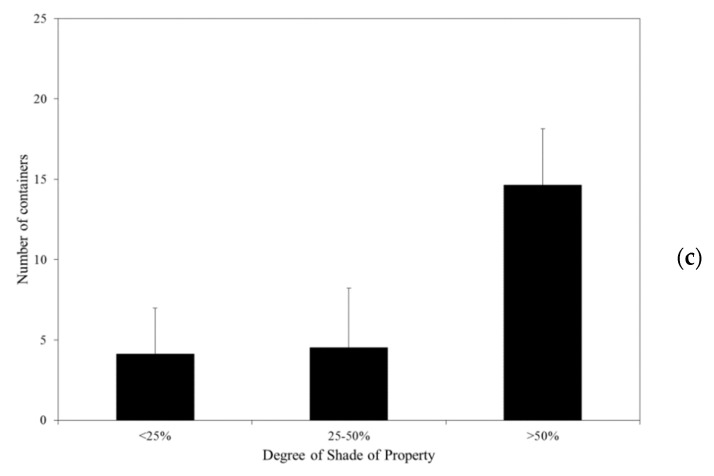
Comparison of mean number of containers per property according to state of property (**a**), condition of property (**b**), and degree of shade (**c**) in the suburb of Carrington, NSW.

When the mean number of containers detected was compared to the three property classifications (Figure 2), there was a statistically significantly higher number of containers recorded from properties classified as untidy (*p* < 0.01; F = 26.72) compared to those classified as tidy or intermediate, and statistically significantly higher number of containers recorded from properties with a higher percentage shade cover (*p* < 0.01; F = 12.11). There was no statistical difference (*p* = 0.07; F = 2.62) in the mean number of containers recorded from the three categories of property condition.

It is important to note that while roof gutters and rainwater tanks are known sources of mosquitoes [41,42], they were generally not surveyed due to difficulty in access and other occupational safety and health concerns. Where possible, rainwater tanks were checked to ensure that screens were in place between down pipes and the main body of the tank but there were very few instances where rainwater tanks were assessed as having faults in installation allowing potential access by mosquitoes. It is worth noting that rainwater tanks were generally uncommon in the area, with less than 2% of properties containing a rainwater tank. It is likely that the prevalence of rainwater tanks in the area will change over time with ongoing renovation and rebuild of residential dwellings and promotion of water-sensitive urban design programs by local authorities.

## 4. Discussion

This study represents the first survey of actual and potential mosquito habitats within a suburb of Newcastle, NSW, adjoining an international port of entry with risk of exotic mosquito incursion. While the above average temperatures and dry conditions were not conducive for local mosquito populations, the results of this survey indicate that there were abundant opportunities for endemic and exotic container-inhabiting mosquito breeding within residential properties adjacent to one of the major NSW shipping ports. Notwithstanding these residential properties, there were industrial properties not included in this survey and these are similarly expected to provide opportunities for container-inhabiting mosquitoes also. Should exotic mosquitoes, primarily *Ae. aegypti* or *Ae. albopictus*, arrive on vessels, there would be opportunities for their establishment in the local area should an incursion occur. Given the substantial pest and public health threats posed by these mosquitoes [6,43], there is a critical need for local authorities to develop response plans and build capacity to rapidly respond to incursions of exotic mosquitoes [3] and the outcomes of this investigation suggest that these plans must consider the operational difficulties in implementing surveillance and exotic mosquito eradication programs.

Surveys of residential properties in many parts of Australia suggest that the findings of the current study strongly align with those undertaken in Brisbane, QLD [39], and Perth, WA [21,44], where a high abundance of water-holding containers were identified as providing suitable conditions for *Ae. notoscriptus* but variability across individual properties in the areas of interest should be expected. Surveys in a suburban area of Brisbane, QLD, identified *Ae. notoscriptus* as the dominant mosquito collected as larvae from water-holding containers, with natural water-holding capabilities (e.g., water-holding plants, water-filled tree holes), garden accoutrements, rubbish, and discarded household items being the most frequently found containing immature mosquitoes (39). Similarly, surveys of properties around Cairns, QLD, found *Ae. notoscriptus*, together with *Ae. aegypti*, in a wide variety of container types [42]. There may also have been subterranean habitats, particularly those associated with stormwater or other infrastructure known to be potential mosquito habitats [45], that were not identified in this study and these should also be considered as sources of mosquitoes beyond those reported here.

There are indices developed to assess the characterisation and productivity of habitats within urban settings associated with abundance of container-inhabiting mosquitoes, particularly *Ae. aegypti* [19,38,46,47]. The adverse environmental conditions during this study that resulted in few water-filled containers being surveyed and the lack of data on abundance of immature stages of *Ae. notoscriptus* prohibited reliable application of these measures to endemic container-inhabiting mosquitoes. 

The condition of properties and associated dwellings has been identified as an indicator of *Ae. aegypti* activity that can assist mosquito surveys by focusing attention on higher risk properties [48]. The results of the current investigation demonstrate that there is considerable variability in the presence and abundance of water-holding containers across the study site, suggesting that any response to the detection of exotic container-inhabiting mosquitoes, such as *Ae. aegypti* or *Ae. albopictus*, will require exhaustive efforts to ensure comprehensive surveillance and control. While the condition of a property may be expected to be a key identifier for high-risk properties (i.e., actual and potential water-filled containers more likely in poorly maintained properties), it was found that even within clean and tidy properties, habitats suitable for endemic, and potentially exotic, container-inhabiting mosquitoes were present. While, as it may be expected, there were more containers found in properties classified as untidy, “street front profiling” of high-risk properties is unlikely to provide a surveillance short cut in response to an incursion of an exotic mosquito. It is worth noting that the factors relating to property type and associated container type prevalence reported here are unique to the setting of the suburb of Carrington, Newcastle, but it would be expected that comparable studies in other areas of NSW would be expected to identify a greater diversity of both property types and associated actual and potential water-holding containers. However, despite the highly focused nature of the current study, the implications for identifying high-risk properties in other regions remain relevant.

There will be property specific factors that determine suitability for exotic mosquitoes. Based on the results of this investigation, it will be difficult to gain access to many properties to conduct surveys, especially during business hours. It is highly likely that property surveys will be required outside normal business hours and on weekends. Given that it took a minimum of 10–12 min to inspect each property, should a response to an exotic mosquito incursion be required that includes disposal or emptying of water-holding containers or chemical treatment, it is likely to require substantial resources. There will be a critical need to actively engage the local community for their assistance in reducing opportunities for mosquitoes, as has successfully been demonstrated in North America [49], but given that there remain substantial gaps in community understandings of mosquito biology and habitats [21,50], considerable effort will be required to raise awareness and understanding of pest and public health risk and required actions.

The greatest risk for the Port of Newcastle is that a vessel arrives carrying water-holding containers supporting unhatched eggs or larvae of exotic mosquitoes. Additionally, live adult mosquitoes may be present on the vessel. Mosquitoes may disperse from berthed vessels and seek out refuge in nearby habitats but the risk of successful incursion of an exotic mosquito will be limited by a number of factors. There has not to date been a case of an exotic mosquito incursion in suburbs around any Australian airport or seaport following detection within the 400 m area of DAWE surveillance. However, there have been detections at freight-handling facilities (i.e., DAWE approved premises) in Sydney and Brisbane. The absence of incursions is testimony to the surveillance and control programs implemented by DAWE, together with local health and airport-managing authorities. Given the increasing rate at which exotic mosquitoes are detected at airports around Australia in recent years, local authorities should be prepared to respond to the detection of an exotic mosquito at the Port of Newcastle. Similarly, if any airports within the local region commence international flights, or direct flights between far north QLD cities (e.g., Cairns, Townsville), consideration will also need to be given to these potential pathways of introduction of *Ae. aegypti*.

It is also worth noting that *Cx. quinquefasciatus*, as well as another species, *Culex pipens molestus* (Forskal) (Diptera: Culicidae), recorded from Newcastle and surrounding suburbs is not a native species. *Culex quinquefasciatus* is one of the most widespread mosquitoes in the world and is likely to have arrived in Australia with European settlers [27]. *Culex molestus* is thought to have been introduced into Victoria in the late 1940s and subsequently spread across urban areas of southern Australia [51]. These two mosquitoes are typically associated with urban freshwater environments, commonly found in polluted stormwater and wastewater infrastructure and water-holding containers in residential properties. The detection of immature stages of *Cx. quinquefasciatus* in this survey was not unexpected, and the larvae of this mosquito are commonly found in water-holding container surveys [21,39], but it is highly likely that *Cx. molestus* is present in the suburb, given the close association of immature stages with subterranean habitats [51]. While the focus of this investigation was on the potential risk associated with *Ae. aegypti* and *Ae. albopictus*, other exotic mosquitoes also pose a risk and should be considered, this includes those associated with water-holding containers and other waterbodies (e.g., stormwater infrastructure, wetlands).

While there were an abundance of available potential habitats for container-inhabiting mosquitoes identified in this study, the receptiveness of the local environment to exotic mosquitoes must also be assessed against a number of other criteria. The property surveys clearly identified opportunities for exotic mosquitoes to seek refuge and lay eggs in receptive water-holding containers, especially water-holding plants, garden accoutrements, and other discarded household items that would be expected to be continually present in the local area. Notwithstanding the presence of suitable natural or artificial water-holding containers, climate is a critical factor in determining the receptiveness of the local environment to exotic mosquito establishment following an incursion event. Rainfall, humidity and day length (i.e., photoperiod) all play a role in providing suitable conditions for exotic mosquitoes [30,52,53,54]. As was the case during February and March 2016 in the lead up to the house-to-house surveys, the higher than average temperatures and below average rainfall resulted in generally unsuitable conditions for mosquitoes overall. While it would have been desirable to undertake a survey of this nature during more favorable climatic conditions, that was not possible due to limited resources. For the planning by local authorities, it is highly unpredictable whether climatic conditions during an incursion event will match those of property surveys of this nature but, as demonstrated in this study, key aspects of the receptibility of local habitats to container-inhabiting mosquitoes have been documented and form a useful base for future strategic response planning. There is also some uncertainty regarding the extent of future climate change in the local region and the implications for establishment of exotic mosquitoes [26] but given that *Ae. aegypti* has been present in the region in the past [28], it should be expected that conditions are likely to become more, rather than less, suitable over time.

While increasing temperatures are expected for the local region in conjunction with a changing climate, rainfall patterns may be less reliable [55]. It was beyond the scope of this investigation to assess local climate variability in providing suitable conditions within the study site for *Ae. aegypti* or *Ae. albopictus* but prevailing climatic conditions will be a critical consideration when assessing the suitability of local environments for establishment of mosquito populations following incursion and associated response. Historically, *Ae. aegypti* was present in the Newcastle region and up until the 1950s, populations of *Ae. aegypti* were common in urban areas along the east coast, with local transmission of dengue thought to have occurred around the Central Coast of NSW [28]. When the global distribution of *Ae. aegypti* is considered, Newcastle would appear to fall within a receptive zone and it is still unclear what influenced the dramatic change in the Australian distribution of *Ae. aegypti*; there are likely to be multiple explanations [28]. The environmental conditions around Newcastle are likely to be suitable to support at least seasonal activity of this mosquito but conditions may not necessarily be suitable for overwintering of the mosquito. While there has not been a specific survey for *Ae. aegypti* undertaken in the Newcastle region, there have been extensive mosquito surveys in the surrounding region, in addition to the Port of Newcastle, and no records of specimens have been found, so it is highly unlikely that remnant populations of the mosquito exist in the local area. Surveys in far north NSW in the 1980s and 2000s also failed to detect any *Ae. aegypti* in local habitats [56,57]. Based on available data, it is reasonable to conclude that NSW does not have any resident populations of *Ae. aegypti* but that should not be interpreted as local conditions not being suitable for the establishment of this mosquito locally.

For populations of *Ae. aegypti* or *Ae. albopictus* to become established in the local area, there would most likely need to be an incursion between the months of November and April, with suitable temperature, rainfall and humidity to facilitate adequate survival of the mosquito to obtain a blood meal and lay eggs in a suitable habitat. The seasonal climate of Newcastle can be variable so that there may be seasonal shifts in the potential for exotic mosquitoes to become established. However, based on the results of this survey, there are well-shaded and humid conditions combined with an abundance of suitable water-holding containers, which are likely to be conducive for mosquito establishment. Current endemic container-inhabiting mosquitoes, such as *Ae. notoscriptus*, are unlikely to pose competitively adverse ecological conditions [58,59]. Low rainfall may not necessarily be a barrier to establishment either as rainwater tanks and other domestic water storages concomitant with a changing climate would provide suitable conditions [29,41]. These conditions for the establishment of these mosquitoes are likely to be enhanced during periods of above average rainfall and temperature.

A number of studies have assessed the suitability of temperate regions for the establishment of *Ae. albopictus* including Europe and North America [60,61]. There are a number of constraints on the predicted spread and invasiveness of this mosquito in these regions, primarily determined by temperature, rainfall and photoperiod. Care must be taken in applying these predictions to NSW given the subtle changes of maximum and minimum temperatures, together with rainfall, that may occur each season. While Asian strains of *Ae. albopictus* are unlikely to survive the winter, North American strains of *Ae. albopictus* may have a higher likelihood of survival should they be introduced. The mosquito is able to persist during the cooler months through diapausing eggs but studies have indicated variability in overwintering survivorship of eggs of different geographic strains of the mosquitoes but there are also some discrepancies between the findings of laboratory studies and experiences in the field. This has been complicated further by evidence of adaptive evolutionary responses in populations of *Ae. albopictus* in North America, with the mosquito increasing over wintering success in colder climates [61]. It is important that should an incursion of *Ae. albopictus* occur late in the season, even if adult mosquitoes are not detected, surveys will be required with the onset of warmer weather in spring as eggs laid during the late summer and early autumn will successfully overwinter and may hatch in spring.

Considering the risk of local exotic pathogen transmission, there would need to be a well-established population and abundance of suitable mosquitoes in Newcastle to create an elevated risk of local transmission of DENV, CHIKV or ZIKV. Even in QLD, where populations of *Ae. aegypti* are present, there are few, if any, local outbreaks of DENV with an overall risk low to moderate [37]. Further assessment requires an understanding of not only the local mosquitoes and their abundance but pathways of infected individuals into the local area and diagnosis of infection. The threat of local transmission resulting from imported cases of DENV may require a suite of control strategies to be employed [62] and at great financial and operational burden to local authorities [6]. It is highly unlikely that an infected mosquito will disperse from a vessel into nearby suburbs and infect local residents. There have been recent examples of “airport dengue” in the Northern Territory, where an infected mosquito thought to have arrived in an aircraft infected an individual working in an industrial area adjacent to Darwin airport [63]. Similarly, a case of DENV was reported in a resident without overseas travel that was suspected to have been caused by an infected mosquito arriving from overseas in an aircraft and transported in personal belongings [64]. There is likely to be a lower risk of infected mosquitoes arriving with vessels at the Port of Newcastle but with the potential that crew, infected prior to departure, are viraemic during travel, there is a small possibility that infected mosquitoes may be present on arrival. With this suburb of Newcastle, well within the typical flight range of *Ae. aegypti* and *Ae. albopictus*, there remains a risk of an infective mosquito transmitting an exotic mosquito-borne pathogen to a local resident. 

The focus of this study was on *Ae. aegypti* and *Ae. albopictus,* two species that should remain a priority for local authorities. However, it is important to note that a suite of mosquito species have been identified in recent years that require careful consideration with regard to invasiveness in temperate climate regions of Australia. *Aedes japonicus* (Theobald) (Diptera: Culicidae), *Aedes koreicus* (Edwards) (Diptera: Culicidae), and *Aedes flavopictus* (Yamada) (Theobald) (Diptera: Culicidae) are just three invasive mosquito species that have been identified as possible incursion risks within temperate regions of the world that may bring with them increased pest and public health concerns [65,66,67,68]. Importantly, there is growing evidence that there are interactions between invasive mosquitoes and endemic mosquitoes that share immature habitats that may be additionally influenced by a changing climate [69,70,71]. It is likely that the pathways of introduction, risk of incursion, and establishment of these invasive mosquitoes will required ongoing review.

While existing surveillance programs are effective at detecting changes in the abundance of local mosquitoes and endemic mosquito-borne pathogens in humans, new strategies are required to monitor exotic mosquitoes in NSW [3]. Local authorities coordinate mosquito and arbovirus surveillance in NSW through the use of carbon dioxide-baited light traps placed within the interface between people and local environments (i.e., wetlands and bushland). This surveillance provides data on relative mosquito abundance and activity of arboviruses, assisting public health risk assessment and informing public health communications from local authorities with regard to the use of effective personal protection measures to avoid mosquito bites. While effective in providing surveillance for endemic mosquitos and mosquito-borne pathogens, this approach is less likely to be effective at detecting an incursion of an exotic mosquito species, especially within urban areas of the region. Notwithstanding the preference for trap locations to be placed at the margins of residential or industrial areas, the types of traps used are generally considered less effective at detecting these exotic mosquitoes that are predominantly active during the day [3].

It will be critical that authorities look to augment their surveillance approaches to better detect and track exotic mosquitoes. While current mosquito traps utilized in NSW (i.e., carbon dioxide-baited light traps) can collect exotic mosquitoes, there is a range of other surveillance technologies that can more effectively key mosquitoes such as *Ae. aegypti* and *Ae. albopictus*. Surveillance capacity should be expanded to include equipment including Biogents Sentinel (BGS) Traps and Gravid Aedes Traps (GAT) that have both been demonstrated as effective and a key component of exotic mosquito response strategies elsewhere [13,32,37,72,73]. Notwithstanding the specific mosquito surveillance technologies employed, careful consideration is required to the broader surveillance program in areas such as those adjacent to seaports, airports and approved arrangements (e.g., freight-handling facilities) that may be an entry point for exotic mosquitoes. This study highlights a model for rapid risk assessment of such locations. However, there are financial and operational challenges to local authorities in maintaining comprehensive surveillance programs.

There may be financial and operational barriers to implementing extensive exotic mosquito surveillance outside first ports of entry. There may be potential in incorporating alternative surveillance methods including citizen science-based programs to expand and/or enhance formal surveillance programs. Community led surveillance incorporating affordable mosquito trapping and smartphone technology has been shown to work effectively [74] and the incorporation of molecular techniques to specimen processing may be benefit in rapid detection of exotic mosquitoes [75,76] It is important that nuisance biting reported to local authorities is investigated. The suburbs within the study area are likely to be impacted by mosquitoes dispersing from nearby estuarine wetlands, as major pest species such as *Ae. vigilax* disperse many kilometres [77], but also locally abundant mosquitoes such as *Ae. notoscriptus* will be responsible for nuisance-biting impacts too. An incursion of exotic mosquitoes may be indicated by a change in local pest impacts, particularly given that the nuisance biting of *Ae. albopictus* can be significant [7] and is considered to be far more disruptive due to their propensity to bite aggressively during the day, more so than the nuisance biting of endemic mosquitoes. An integrated approach to exotic mosquitoes is required beyond a simple reliance on formal surveillance programs.

Finally, critical to the success of assessing and responding to an incursion in this area will also be the rapid commencement of house-to-house surveys to assess the extent of incursion and to commence source reduction and control of exotic mosquito populations. A community engagement program will be essential to mitigate anxiety among local residents of immediate public health threat associated with the detection of exotic mosquitoes together with likely resistance to the use of insecticides within properties as well as disturbance to the property through the tipping out of or removal of water-holding containers.

## 5. Conclusions

Exotic mosquitoes, primarily *Ae. aegypti* and *Ae. albopictus*, pose a threat to human health in NSW. The results of this investigation indicate that an abundance of suitable habitats exist within the suburban areas adjacent to the Port of Newcastle, within the known flight ranges of these key mosquitoes. Should an incursion of these mosquitoes occur through arrival on vessels, there would be opportunities for their establishment in the local area. The outcomes of this investigation have clearly demonstrated the need to continue surveillance within first ports of entry including seaports. This study has also highlighted the need to build capacity within local health authorities to understand the risks posed by exotic mosquito incursions and to develop locally relevant response plans should an incursion occur.

## Figures and Tables

**Table 1:** A summary of container types and mosquito species detected in the surveys of 337 properties in Carrington, Newcastle, March 2016.

Container Type	Total Number of Container Types Recorded across All Properties	Number Properties with at Least One Container Type	Mosquito Species Detected as Larvae from at Least One Container Type within at Least One Property
Roof gutters ^1^	204	204	N/A
Rainwater tank ^1^	3	3	N/A
Water barrel	7	3	*Ae. notoscriptus*; *Cx. quinquefasciatus*
Bucket	120	61	*Ae. notoscriptus*; *Cx. quinquefasciatus*
Drain	16	14	*Cx. quinquefasciatus*
Bromeliads	131	17	*Ae. notoscriptus*; *Cx. quinquefasciatus*
Striking bucket	1	1	*Cx. quinquefasciatus*
Vase	3	2	N/A
Bird bath	8	8	*Ae. notoscriptus*
Boat/trailer	4	3	*Cx. quinquefasciatus*
Pot plants	520	116	*Ae. notoscriptus*
Tyre	21	6	*Ae. notoscriptus*
Misc (<2 L)	311	79	*Ae. notoscriptus*; *Cx. quinquefasciatus*
Misc (2–20 L)	84	45	*Ae. notoscriptus*; *Cx. quinquefasciatus*
Misc (>20 L)	29	24	*Cx. quinquefasciatus*
Dog bowl	21	12	N/A
Plastic sheets	9	8	*Ae. notoscriptus*; *Cx. quinquefasciatus*
Discarded toy	9	7	*Ae. notoscriptus*; *Cx. quinquefasciatus*
Frog pond	5	3	*Ae. notoscriptus*; *Cx. quinquefasciatus*

^1^ Habitats not surveyed for immature mosquitoes; N/A—not applicable.
